# Situation and Cercarial Infection of Freshwater Mollusk from Sirindhorn Reservoir, Ubon Ratchathani Province, Thailand

**Published:** 2019

**Authors:** Surat HARUAY, Supawadee PIRATAE

**Affiliations:** 1. Community Health Program, Faculty of Public Health, Ubon Ratchathani Rajabhat University, Ubon Ratchathani, Thailand; 2. One Health Research Unit, Faculty of Veterinary Sciences, Mahasarakham University, Maha Sarakham, Thailand

**Keywords:** Trematode cercariae, Survey, Freshwater snails, Diversity

## Abstract

**Background:**

Most of trematodes need snails to complete their life cycles. Consequently freshwater snails are served as intermediate hosts of many parasites worldwide. There is a lack of report on snail diversity and parasitic infection in snails in Sirindhorn Reservoir, Ubon Ratchathani province, Thailand.

**Methods:**

Mollusk diversity and trematode cercariae infections were investigated in snails from 120 sampling sites surround Sirindhorn Reservoir from April 2018 to June 2018. Mollusk species were identified based on their shell morphology. The presence of cercariae infections in snails was examined by cercarial shedding methods. The interaction between snail species was analyzed by the correlation method.

**Results:**

Overall, 2076 mollusks were collected which comprised six species of snails and two species of bivalves. Snail species were identified as *Bithynia siamensis goniomphalos, Anentome helena, Filopaludina sumatrensis spiciosa*, *F. martensi martensi, F. martensi munensis* and *Pomacea canaliculata.* The overall rate of trematode cercariae infection was 1.69% (35/2,076). The cercariae found infecting snails were Cercariaeum cercaria, Virgulate cercaria, Cotylomicrocercous cercaria and Furcocercous cercaria. The most common snails found was the assassin snail, *A. helena,* which showed the negative relationship among other species interactions.

**Conclusion:**

This finding indicated infection with animal's parasites in snails in this area are common, besides, we found many species of snails in Sirindhorn Reservoir are potentially be the host of parasite in animal and human.

## Introduction

Infection with trematode parasite remains medically importance problem worldwide especially in neglected countries (1,2). To complete the trematode parasites life cycle, snails are required to be the intermediate host. In snail host, the trematode eggs can hatch in the environment to be miracidia which can penetrate into snail intermediate hosts (3) or eggs can hatch within the gastrointestinal tract of the snails (4). After the miracidia infection, they can develop and transform to be sporocyst, redia and cercaria stages. The cercaria will be released from infected snails for finding the new host or transformation to be metacercaria stage in further. Currently, quickly increased snails number are emerging problems worldwide, especially in the area where a dam is situated since they can spread extensively snail borne parasitic diseases (5–7). Although infection rate of snails is very low, they can release a numerous number of the parasite larva (8). Accordingly, the study of epidemiology of snails and their infected cercariae is extra essential and need to be concern.

In Thailand, the studies related to snails diversity and cercarial infection are insufficient (9–11), especially in Sirindhorn Reservoir where is an important area used for agricultural purposes, animal husbandry, also the source of fish farming. No study has been surveyed snail diversity and parasitic infection in snail in Sirindhorn Reservoir, Ubon Ratchathani Province, Thailand. This study surveyed the occurrence and distribution of snails and their trematode infections using cercarial shedding methods in 120 stations in Sirindhorn Reservoir, Ubon Ratchathani Province, Thailand.

## Materials and Methods

### Study area and sample collection

**Fig. 1:**
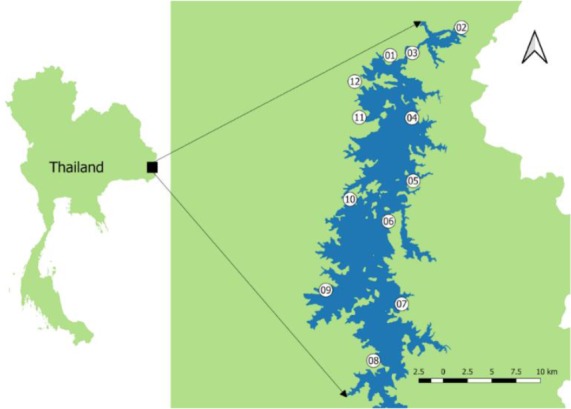
Map of 12 localities in Sirindhorn Reservoir, Ubon Ratchathani province, Thailand where mollusk samples were collected

The freshwater mollusks were collected during Apr 2018 to Jun 2018 which is overlap between summer season and rainy season, temperature ranging from 24–36 °C and the annually rainfall is 1600–1800 mm (Thai Meteorological Department, 2017). Samples were collected from 12 localities (1 locality consists of 10 stations where were 10–15 meters far from each other) surround Sirindhorn Reservoir, Ubon Ratchathani province of Thailand (Fig. 1). Mollusks were collected by hand for a period of 5 min/station per trained person (12,13) from the coast of the reservoir where the waterbed is sandy, soil, mud and rocks. Mollusks were kept in plastic bags with holes during transport to the laboratory for further study. Mollusks were identified based on shell and operculum morphology (14,15).

All experimental procedures using animals were approved by the Animal Care and Use Committee, Ubon Ratchathani Rajabhat University (AE612008).

### Examination of cercarial infection in snails

To examine trematode infection in snails, cercarial shedding method was performed according to the previous study (16). Briefly, snails were cleaned to remove mud and plant materials with dechlorinated tap water. Each snail was measured, placed in a small cup (3 cm in diameter and 2.5 cm in high) containing 10 ml of dechlorinated tap water. For daytime, shedding was performed by turn on an electric light for 3 hours to induce releasing of cercariae from the infected snails. For nighttime, cups of snails were kept in the darkness overnight. Cercarial shedding was performed both daytime and nighttime and observed cercariae of the trematodes under the stereomicroscope (13–17). The presence of cercariae was identified based on their morphology and movement under the stereomicroscope by identification key (18). Non-infected snails were delivered back to the Sirindhorn Reservoir.

### Data analysis

Snail size was expressed as the mean ± standard error. The infection rate was expressed as percentage. The interaction between snail species was analyzed. Correlation of species interaction was performed using the R project for Statistical Computing (https://www.r-project.org/). Statistically significant was defined as *P* less than 0.05.

## Results

### Situation of mollusk in Sirindhorn reservoir

**Fig. 2:**
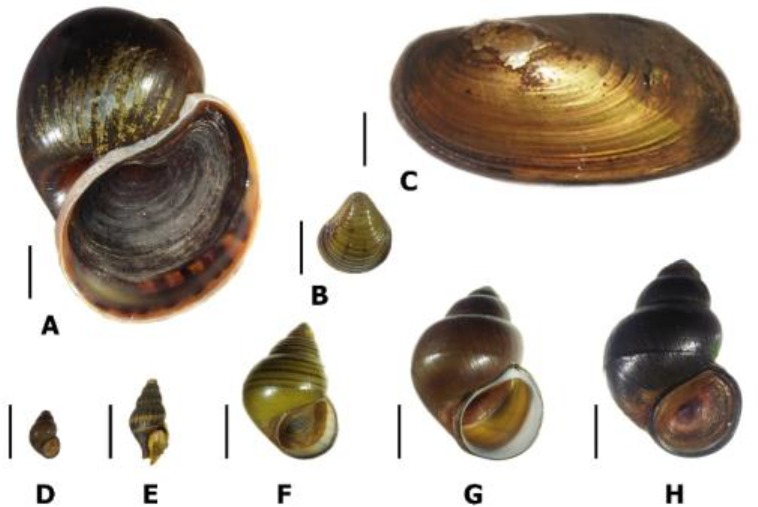
Shell morphology of six snails and two bivalves in Sirindhorn Reservoir. **A**= *Pomacea canaliculata*; **B**= *Corbicula* sp.; **C**= *Pilsbryoconcha exilis*; **D** = *Bithynia siamensis goniomphalos*; **E** = *Anentome helena*; **F** = *Filopaludina sumatrensis spiciosa*; **G** = *Filopaludina martensi munensis*; **H** = *Filopaludina martensi martensi*. Scale bar: A–H = 1 cm

The present study represents the first report on species diversity and cercariae trematode infection in snails in Sirindhorn Reservoir. A total of 2,076 mollusks collected were classified into 4 families, 4 genus and 6 species of gastropod snails: the Nassariidae (*Anentome helena*), Bithyniidae (*Bithynia siamensis goniomphalos*), Viviparidae (*Filopaludina sumatrensis spiciosa, F. martensi martensi, F. martensi munensis*) and Ampullariidae (*Pomacea canaliculata*) in addition 2 families, 2 genus and 2 species of bivalves: Cyrenidae (*Corbicula* sp.) and Unionidae (*Pilsbryoconcha exilis*) (Fig. 2).

In total, 2,000specimens belonging to snails, 53.66% (1,114/2,076) were the assassin snail, *A. helena*, 16.57%(344/2,076) were *F. s. spiciosa*, 12.67% (263/2,076) were *B. s. goniomphalos*, 10.55% (219/2,076) were *F. m. munensis,* 2.36% (49/2,076) were *F. m. martensi* and 0.53% (11/2,076) were *P. canaliculata.* Additionally, among 76 specimens of bivalves, 3.37% (70/2,076) were *Corbicula* sp. and 0.29% (6/2,076) were *P. exilis* (Table 1).

**Table 1:** Species and number of freshwater mollusk collected from 12 localities from Sirindhorn Reservoir, Ubon Ratchathani Province, Thailand during Apr 2018 to Jun 2018

	***Locality / Latitude, Longitude***	Number of Snails^*^								***Total***
		***Bsg***	***Ah***	***Fs***	***Fma***	***Fmu***	***Pc***	***Csp***	***Pe***	
1	15°10′54″N 105°23′34″E	-	43	111	6	-	6	16	-	182
2	15°12′20″N 105°27′42″E	66	52	14	4	54	-	-	-	190
3	15°11′ 0″N 105°24′51″E	-	68	14	11	15	-	-	-	108
4	15° 7′23″N 105°24′43″E	22	201	59	13	-	1	3	-	299
5	15° 3′52″N 105°24′40″E	3	135	1	5	6	1	1	-	152
6	15° 1′40″N 105°23′12″E	-	133	-	2	2	-	8	-	145
7	14°57′ 4″N 105°23′47″E	-	11	1	-	42	-	9	5	68
8	14°54′40″N 105°22′29″E	-	37	4	-	56	-	15	1	113
9	14°58′34″N 105°20′ 0″E	-	75	13	-	44	1	6	-	139
10	15° 2′42″N 105°20′53″E	-	222	-	2	-	-	7	-	231
11	15° 7′30″N 105°21′42″E	27	76	1	2	-	2	1	-	109
12	15° 9′30″N 105°21′30″E	145	61	126	4	-	-	4	-	340
	No. of Total (%)	263 (12.67)	1,114 (53.66)	344 (16.57)	49 (2.36)	219 (10.55)	11 (0.53)	70 (3.37)	6 (0.29)	2,076

Number of Snails

* : Bsg = *Bithynia siamensis goniomphalos*; Ah = *Anentome helena*; Fs = *Filopaludina sumatrensis spiciosa*; Fma = *Filopaludina martensi martensi*; Fmu = *Filopaludina martensi munensis*; Pc = *Pomacea canaliculata*; Csp = *Corbicula* sp.; Pe = *Pilsbryoconcha exilis*

### Trematode cercariae infection

**Fig. 3:**
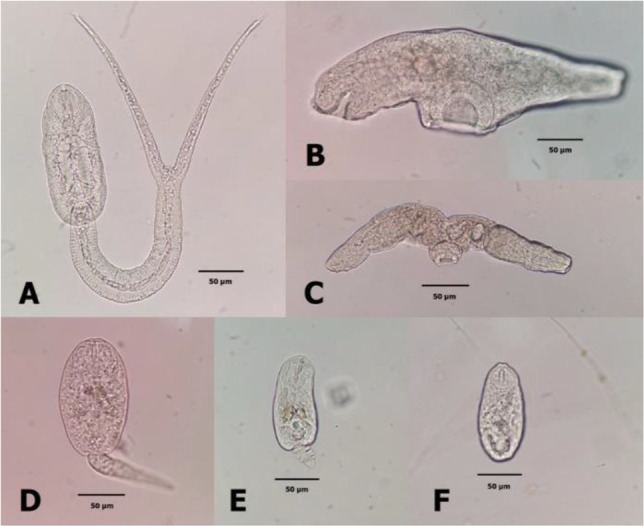
Cercariae types in snails: **A**= Furcocercous cercaria (*A. helena*); **B**= Cercariaeum cercaria (*B. s. goniomphalos*); **C**= Cotylomicrocercous cercaria (*A. helena*); **D** = Virgulate cercaria (*F. s. spiciosa*); **E** = Virgulate cercaria (*A. helena*); **F** = Virgulate cercaria (*B. s. goniomphalos*)

Three species of snails namely, *B. s. goniomphalos*, *A. helena* and *F. s. spiciosa* were found as the intermediate host of cercariae which were Cotylomicrocercous cercaria, Virgulate cercaria, Cercariaeum cercaria and Furcocercous cercaria (Fig. 3). The infection rate of Virgulate cercaria was 4.18% in *B. s. goniomphalos*, 0.09% in *A. helena* and 0.29% in *F. s. spiciosa.* The infection rate of Cercariaeum cercaria were 0.38% in *B. s. goniomphalos*. Snail *A. helena* were infected with 1.80% of Cotylomicrocercous cercariae and 0.09% of Furcocercous cercariae (Table 2).

**Table 2:** Cercariae infection rate of snails from Sirindhorn Reservoir, Ubon Ratchathani province, Thailand

***Snail species / Type of cercaria***		***Locality / Infection rate (Number of infected snails)***						***Total Infection rate***
		***2***	***4***	***5***	***6***	***10***	***12***	
*B. s. goniomphalos*	Virgulate cercaria	4.55% (3/66)	4.55% (1/22)	-	-	-	4.83% (7/145)	4.18% (11/263)
	Cercariaeum cercaria	-	-	-	-	-	0.69% (1/145)	0.38% (1/263)
*A. helena*	Cotylomicrocercous cercaria	-	5.47% (11/201)	0.74% (1/135)	6.02% (8/133)	-	-	1.80% (20/1,114)
	Furcocercous cercaria	-	-	-	-	0.45% (1/222)	-	0.09% (1/1,114)
	Virgulate cercaria	-	-	-	-	0.45% (1/222)	-	0.09% (1/1,114)
*F.s. spiciosa*	Virgulate cercaria	-	-	-	-	-	0.79% (1/126)	0.29% (1/344)
Total infection		1.58% (3/190)	4.01% (12/299)	0.66% (1/152)	5.52% (8/145)	0.87% (2/231)	2.65% (9/340)	1.69% (35/2,076)

### Relationship between species interactions and Assassin snail abundance

**Fig. 4:**
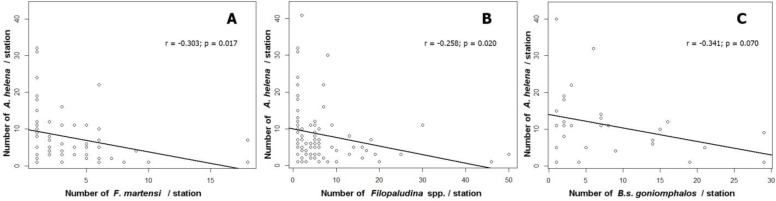
Relationship between assassin snail, *Anentome helana,* abundance and species interactions: A: Negative correlation with *F. martensi* (*r* = −0.303; *P*<0.05); B: Negative correlation with *Filopaludina* spp. (*r* = −0.258; *P*<0.05); C: Negative correlation on density with *B. s. goniomphalos* (*r* = −0.341; *P*=0.07)

The relationship between species interactions and *A. helena* (assassin snail) richness were analyzed. The relative abundance of *A. helena* was statistically negative correlation with the presence of *F. martensi* (*r* = −0.303; *P*<0.05) and *Filopaludina* spp. (*r* = −0.258; *P*<0.05). Moreover, the species richness was found negative correlation trend between *A. helena* and *B. s. goniomphalos* (*r* = −0.341; *P*=0.07) but no statistically relationship (Fig. 4).

## Discussion

Several research have reported mollusk diversity and their potential roles to be the intermediate host of the parasites (19–20). In this study, we found six species of snails, some of them have been reported as the host of parasite namely, *B. s. goniomphalos, Filopaludina* sp. and *P. canalicalata.* Snail *B. s. goniomphalos* is the first intermediate host of a human liver fluke, *Opisthorchis viverrini*, the infected snail can release cercariae to infect cyprinoid fish in the next step (21–22). Snail *Filopaludina* sp. is the host of Echinostomes intestinal fluke, the infected snails able to be the second intermediate host of metacercariae (23). Snail *P. canalicalata* is the intermediate host of the rat lungworm, *Angiostrongylus cantonensis* (24–25). Our finding indicated that some of snail species in this area can potentially be host of medical parasites.

From cercarial examination, four type cercariae were found which are Cotylomicrocercous cercaria (parasite of fish), Virgulate cercaria (intestinal parasites of birds and amphibians), Cercariaeum cercaria (intestinal parasites of fish and parasites of the respiratory tract of birds) (18) and Furcocercous cercaria (blood parasite of vertebrate animals). The total infection rate of cercariae in Sirindhorn reservoir was 1.69% (35/2,076) which lower than the reported of cercarial infections in snails in Chao-Phraya Basin (5.90%) (9) Chiang Mai province, Thailand (17.27%) (10) and Nakhon Nayok Province, Thailand (4.7%) (11), these may due to the difference of water reservoirs and snails diversity in each water area.

In Sirindhorn reservoir, snail *F. s. spiciosa* were infected with Virgulate cercariae (0.29%) but not found infection of this snail in Nakhon Nayok Province, Thailand (11). *B. s. goniomphalos* in Sirindhorn reservoir is infected with Virgulate cercaria (4.18%) which supported the previous report regarding the presence of Virgulate cercaria in this snail (13–17). Moreover, this snail is infected with Cercariaeum cercaria (0.38%) which has not been reported in any study in Thailand. Besides, our study found no bithyniid snail infected with human liver fluke, *Opisthorchis viverrini*, supporting to the study of metacercaria in fish in this area which found only 4 metacercaria of *O. viverrini* (observed from 840 cyprinoid fish) (26) indicated that infection rate in the intermediate host is relatively low due to Ubon Ratchathani is an endemic area of opisthorchiasis where the infection rate in human is 14.8% (27).

In addition, we found *A. helena* served as the intermediate host of Cotylomicrocercous cercariae, Furcocercous cercariae and Virgulate cercariae which different across Chao-Phraya Basin (9) Nakhon Nayok Province, Thailand (11) where *A. helena* snail did not reveal any infections. However, our finding supported the study of trematode infection in *Clea* which found *Clea* collected from four provinces (Nakhon Ratchasima, Buri Ram, Surin and Si Sa Ket) of Thailand are infected with 1.92% of at least one types of Furcocercous cercariae*,* Cotylomicrocercous cercariae and Cercariaeum cercariae (28).

In many countries, freshwater snails have been surveyed and examined for parasitic infections such as Iran (29), Sri Lanka (30), Sudan (31) and Zimbabwe (32). A total of 27.9%, 16%, 14.1% and 6.6% of collected snails from Iran, Sri Lanka, Sudan and Zimbabwe, respectively, released one or more types of cercariae. The difference of cercarial types and infection rates of snails in each area may as a result of the difference of water habitat, endemic area of parasites, species diversity of snail, including the Anthropocene alteration of water environments.

**Fig. 5:**
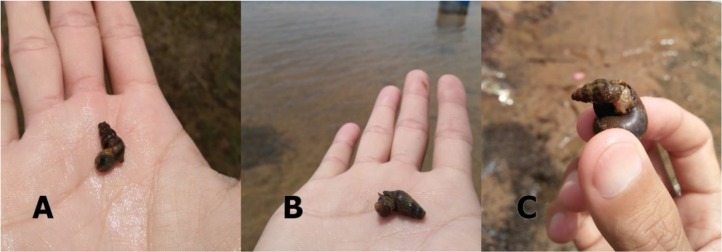
*A. helena* were consuming *Corbicula* sp. **(A)**, *B. s. goniomphalos* **(B)** and *F. s. speciosa* **(C)**

In this study, *A. helena* was the dominant species of snails found in every localities sampling. The highest number of *A. helena* snails were found in areas with sandy ground followed soil, mud and rock which according to the presence of *B. s. goniomphalos*. In contrast, *F. martensi* and *F. s. spiciosa* were preferred muddy area. When analyzed the relationship between species interaction, we found negative correlation between assassin snails (*A. helena)* and *Filopaludina* spp. (*P* < 0.05) also *B. s. goniomphalos* (*P* = 0.07). This finding may be occurred from character of predator of assassin snails, preference habitat and food. *A. helena* has recently been reported as the biological predator which consumed *Melanoides tuberculata* and *Tarebia granifera* snails in a large amount (33), but in the presence of organic residues, the intensity of consumption of *M. tuberculata* and *T. granifera* snails by assassin snails is significantly reduced (34). Due to the ability of mollusks predatory, *A. helena* targeted by the freshwater ornamental pet trade as a result of its predation abilities on other snail species (35). Moreover, as we were sampling mollusks, we found *A. helena* were consuming *Corbicula* sp., *B. s. goniomphalos* and *Filopaludina* spp. (Fig. 5), this event supported the data of future snail-control strategies by using assassin snails.

## Conclusion

Species diversity of snails surround Sirind-horn Reservoir, Ubon Ratchathani province were observed. This study provides data of snail diversity which some of them have been reported as intermediate host of medical parasites and types of infected cercariae in snails which are important for the veterinary and public health.

Furthermore, we also observed the relationship between densities of assassin snails, *A. helena*, and other competitive snails and found negative correlation trends which need for extra study whether *A. helena* can act as the predator of medical mollusk. And whether using *A. helena* to consume other snails can get positive results in controlling snail-borne parasitic diseases or not.

## References

[1] Andrews RH Sithithaworn P Petney TN. *Opisthorchis viverrini*: an underestimated parasite in world health. Trends Parasitol. 2008;24(11):497–501. 18930439 10.1016/j.pt.2008.08.011 PMC2635548

[2] Chai JY Murrell KD Lymbery AJ. Fish-borne parasitic zoonoses: status and issues. Int J Parasitol. 2005;35(11–12):1233–54. 16143336 10.1016/j.ijpara.2005.07.013

[3] Kalbe M Haberl B Haas W. Miracidial host-finding in *Fasciola hepatica* and *Trichobilharzia ocellata* is stimulated by species-specific glycoconjugates released from the host snails. Parasitol Res. 1997;83(8):806–12. 9342748 10.1007/s004360050344

[4] Adam R Pipitgool V Sithithaworn P Hinz E Storch V. Morphology and ultrastructure of the digestive gland of *Bithynia siamensis goniomphalus* (Prosobranchia: Bithyniidae) and alterations induced by infection with the liver fluke *Opisthorchis viverrini* (Trematoda: Digenea). Parasitol Res. 1995;81(8):684–92. 8570585 10.1007/BF00931847

[5] Bass CS Weis JS. Increased abundance of snails and trematode parasites of *Fundulus heteroclitus* (L.) in restored New Jersey wetlands. Wetlands Ecol Manage. 2008;16(3):173–82.

[6] Fenwick A. Waterborne infectious diseases - could they be consigned to history?. Science. 2006;313(5790):1077–81. 16931751 10.1126/science.1127184

[7] Sokolow SH Jones IJ Jocque M Nearly 400 million people are at higher risk of schistosomiasis because dams block the migration of snail-eating river prawns. Philos Trans R Soc Lond B Biol Sci. 2017;372(1722):20160127. 28438916 10.1098/rstb.2016.0127 PMC5413875

[8] Piratae S. *Bithynia siamensis goniomphalos*, the first intermediate host of *Opisthorchis viverrini* in Thailand. Asian Pac J Trop Med. 2015;8(10):779–83. 26522291 10.1016/j.apjtm.2015.09.002

[9] Anucherngchai S Tejangkura T Chontananarth T. Epidemiological situation and molecular identification of cercarial stage in freshwater snails in Chao-Phraya basin, Central Thailand. Asian Pac J Trop Biomed. 2016;6(6):539–45.

[10] Chontananarth T Wongsawad C. Epidemiology of cercarial stage of trematodes in freshwater snails from Chiang Mai province, Thailand. Asian Pac J Trop Biomed. 2013;3(3):237–43. 23620846 10.1016/S2221-1691(13)60058-1 PMC3631758

[11] Chontananarth T Tejangkura T Wetchasart N Chimburut C. Morphological Characteristics and Phylogenetic Trends of Trematode Cercariae in Freshwater Snails from Nakhon Nayok Province, Thailand. Korean J Parasitol. 2017;55(1):47–54. 28285506 10.3347/kjp.2017.55.1.47 PMC5365261

[12] Tesana S. Diversity of mollusks in the Lam Ta Khong reservoir, Nakhon Ratchasima, Thailand. Southeast Asian J Trop Med Public Health, 2002;33:733–8. 12757219

[13] Kulsantiwong J Prasopdee S Labbunruang N Chaiyasaeng M Tesana S. Habitat and trematode infection of *Bithynia siamensis goniomphalos* in Udon Thani province, Thailand. Southeast Asian J Trop Med Public Health. 2017;48(5):975–82.

[14] Brandt RAM. The non-marine aquatic Mollusca of Thailand. Archiv für Molluskenkunde. 1974;105:1–423.

[15] Upatham ES Sornmani S Kitikoon V Lohachit C Burch JB. Identification key for the fresh- and brackish-water snails of Thailand. Malacol Rev. 1983;16:107–32.

[16] Kaewkes S Kaewkes W Boonmars T Sripa B. Effect of light intensity on *Opisthorchis viverrini* cercarial shedding levels from *Bithynia* snails - a preliminary study. Parasitol Int. 2012;61(1):46–8. 21872679 10.1016/j.parint.2011.08.015

[17] Sripa J Kiatsopit N Piratae S. Prevalence of trematode larvae in intermediate hosts: snails and fish in Ko Ae sub-district of Khueang Nai, Ubon Ratchathani province, Thailand. Southeast Asian J Trop Med Public Health. 2016;47(3):399–409. 27405122

[18] Frandsen F Christensen NO. An introductory guide to the identification of cercariae from African freshwater snails with special reference to cercariae of trematode species of medical and veterinary importance. Acta Trop. 1984;41(2):181–202. 6206702

[19] Namchote S Sritongtae S Butnin S Wongwain P Krailas D. Larval stage of trematodes obtained from brackish water snails in the central and east coast of the gulf of Thailand. Sci Res Essays. 2015;10(11):386–401.

[20] Krailas D Namchote S Koonchornboon T Dechruksa W Boonmekam D. Trematodes obtained from the thiarid freshwater snail *Melanoides tuberculata* (Müller, 1774) as vector of human infections in Thailand. Zoosyst Evol. 2014;90:57–86.

[21] Harinasuta C Harinasuta T. *Opisthorchis viverrini*: life cycle, intermediate hosts, transmission to man and geographical distribution in Thailand. Arzneimittelforschung. 1984;34 9B:1164–7. 6542383

[22] Kiatsopit N Sithithaworn P Saijuntha W Exceptionally high prevalence of infection of *Bithynia siamensis goniomphalos* with *Opisthorchis viverrini* cercariae in different wetlands in Thailand and Lao PDR. Am J Trop Med Hyg. 2012;86(3):464–9. 22403318 10.4269/ajtmh.2012.11-0217 PMC3284363

[23] Chai JY Sohn WM Na BK Van De N. *Echinostoma revolutum*: Metacercariae in *Filopaludina* snails from Nam Dinh Province, Vietnam, and adults from experimental hamsters. Korean J Parasitol. 2011;49(4):449–55. 22355218 10.3347/kjp.2011.49.4.449 PMC3279689

[24] Tesana S Srisawangwong T Sithithaworn P Laha T Andrews R. Prevalence and intensity of infection with third stage larvae of *Angiostrongylus cantonensis* in mollusks from Northeast Thailand. Am J Trop Med Hyg. 2009;80:983–7. 19478262

[25] Kim JR Hayes KA Yeung NW Cowie RH. Diverse gastropod hosts of *Angiostrongylus cantonensis*, the rat lungworm, globally and with a focus on the Hawaiian Islands. PloS One. 2014;9 5:e94969. 24788772 10.1371/journal.pone.0094969 PMC4008484

[26] Pinlaor S Onsurathum S Boonmars T Distribution and abundance of *Opisthorchis viverrini* metacercariae in cyprinid fish in Northeastern Thailand. Korean J Parasitol. 2013; 51(6):703–10. 24516277 10.3347/kjp.2013.51.6.703 PMC3916461

[27] Tungtrongchitr A Chiworaporn C Praewanich R Radomyos P Boitano JJ. The potential usefulness of the modified Kato thick smear technique in the detection of intestinal sarcocystosis during field surveys. Southeast Asian J Trop Med Public Health. 2007;38(2): 232–8. 17539271

[28] Yutemsuk N Krailas D Anancharoenkit C Phanpeng L Dechruksa W. Trematode infections of freshwater snails genus *Clea* A. Adams, 1855 in the reservoir of lower northeast Thailand. JITMM Proceedings. 2017;6:7–16.

[29] Kalat-Meimari M Shamseddin J Salahi-Moghaddam A. Ecological and Parasitological Study on *Cerithidea cingulata* (Gastropoda) in Hormoz Strait Littoral, South of Iran. Iran J Parasitol. 2018;13(2): 285–92. 30069213 PMC6068361

[30] Jayawardena U Rajakaruna R Amerasinghe P. Cercariae of trematodes in freshwater snails in three climatic zones in Sri Lanka. Cey J Sci (Bio. Sci.). 2011;39(2):95–108.

[31] Mohammed NA Madsen H Ahmed AAA. Types of trematodes infecting freshwater snails found in irrigation canals in the East Nile locality, Khartoum, Sudan. Infect Dis Poverty. 2016;5:16. 26915911 10.1186/s40249-016-0108-y PMC4766606

[32] Chingwena G Mukaratirwa S Kristensen TK Chimbari M. Larval trematode infections in freshwater snails from the Highveld and Lowveld areas of Zimbabwe. J Helminthol. 2002;76(4),283–293. 12498632 10.1079/JOH2002132

[33] Yakovenko V Fedonenko O Klimenko O Petrovsky O. Biological control of the invasive snail species *Melanoides tuberculata* and *Tarebia granifera* in Zaporizka Nuclear Power Plant cooling pond. Ukr J Ecol. 2018;8(1):975–82.

[34] Oleh M Kyrylo B Olena K. Biological and biomechanical principles of the controlling molluscs *Melanoides tuberculata* (Müller 1774) and *Tarebia granifera* (Lamarck, 1822) in reservoirs of strategic importance. World Sci News. 2018;99:71–83.

[35] Ng TH Foon JK Tan SK Chan MK Yeo DC. First non-native establishment of the carnivorous assassin snail, *Anentome helena* (von dem Busch in Philippi, 1847). Bioinvasions Rec. 2016;5(3):143–8.

